# Hypoxia-Inducible Factor-1α Regulates Chemotactic Migration of Pancreatic Ductal Adenocarcinoma Cells through Directly Transactivating the CX3CR1 Gene

**DOI:** 10.1371/journal.pone.0043399

**Published:** 2012-08-27

**Authors:** Tiansuo Zhao, Song Gao, Xiuchao Wang, Jingcheng Liu, Yitao Duan, Zhanna Yuan, Jun Sheng, Shasha Li, Feng Wang, Ming Yu, He Ren, Jihui Hao

**Affiliations:** 1 Key Laboratory of Cancer Prevention and Therapy, Department of Pancreatic Cancer, Tianjin Medical University Cancer Institute and Hospital, Tianjin, China; 2 The Ministry-of-Education Key Laboratory of System Bioengineering, School of Chemical Engineering and Technology, Tianjin University, Tianjin, China; 3 Institute of Chinese and Modern Medicines for Acute Abdominal Diseases, Tianjin Medical University Nankai Hospital, Tianjin, China; University of Nebraska Medical Center, United States of America

## Abstract

CX3CR1 is an important chemokine receptor and regulates the chemotactic migration of pancreatic ductal adenocarcinoma (PDAC) cells. Up to now, its regulatory mechanism remains largely undefined. Here, we report that hypoxia upregulates the expression of CX3CR1 in pancreatic cancer cells. When hypoxia-inducible factor (HIF)-1α expression was knocked down in vitro and in vivo, the expression of CX3CR1 was significantly decreased. Chromatin immunoprecipitation assay demonstrated that HIF-1α bound to the hypoxia-response element (HRE; 5′-A/GCGTG-3′) of CX3CR1 promoter under normoxia, and this binding was significantly enhanced under hypoxia. Overexpression of HIF-1α significantly upregulated the expression of luciferase reporter gene under the control of the CX3CR1 promoter in pancreatic cancer cells. Importantly, we demonstrated that HIF-1α may regulate cancer cell migration through CX3CR1. The HIF-1α/CX3CR1 pathway might represent a valuable therapeutic target to prevent invasion and distant metastasis in PDAC.

## Introduction

CX3CR1 is normally expressed by hematopoetic cells [1], prostate cancer [2], breast cancer [3] and pancreatic ductal adenocarcinoma (PDAC) [4]. The sole ligand for CX3CR1 is the chemokine CX3CL1, also named Fractalkine/Neurotactin [5], [6]. CX3CL1 is defined as a membrane as well as a soluble chemokine expressed by neurons and activated endothelial cells [7], [8]. Recent evidence has shown that the CX3CL1/CX3CR1 pair plays a major role in adhesion, migration and survival of tumor cells including pancreatic cancer cells [4].

Despite diagnostic and therapeutic advances, PDAC still has a very poor prognosis. PDAC accounts for the fourth largest cause of cancer-related deaths in the United States, and its 5-year survival rate is only 5% [9]. Neuropathic pain is a common phenomenon in PDAC patients [10]. It is well known that tumor neurotropism is a major cause of recurrence after curative resection in PDAC [11]. Although the role of CX3CR1 in the neurotropism of pancreatic cancer has been established, the regulatory mechanism of this chemotactic migration remains to be elucidated.

It is well known that the expression of cytokines is usually regulated by specific transcription factors. Hung et al. [12] reported that hypoxia modified the expression of CX3CR1 in multipotent stromal cells. Previous studies have shown that hypoxia-inducible factors (HIFs) are important in the regulation of hypoxia-related genes [13]. HIF transcription factors consist of highly regulated HIF-1α and HIF-2α subunit and a constitutively expressed HIF-1β subunit. By screening genomic DNA fragments of the human CX3CR1 gene 5′-flanking regions, we found eight hypoxia response elements (HREs), the DNA binding sites of HIFs. Based on these, we postulate that CX3CR1 may be a potential target of HIFs in PDAC.

In this study, we aimed to investigate (i) the mechanism of CX3CR1 regulation by hypoxia, (ii) the role of HIF/CX3CR1 in the chemotactic migration of PDAC, and (iii) the correlation between HIF and CX3CR1 in specimens of pancreatic cancer.

**Figure 1:**
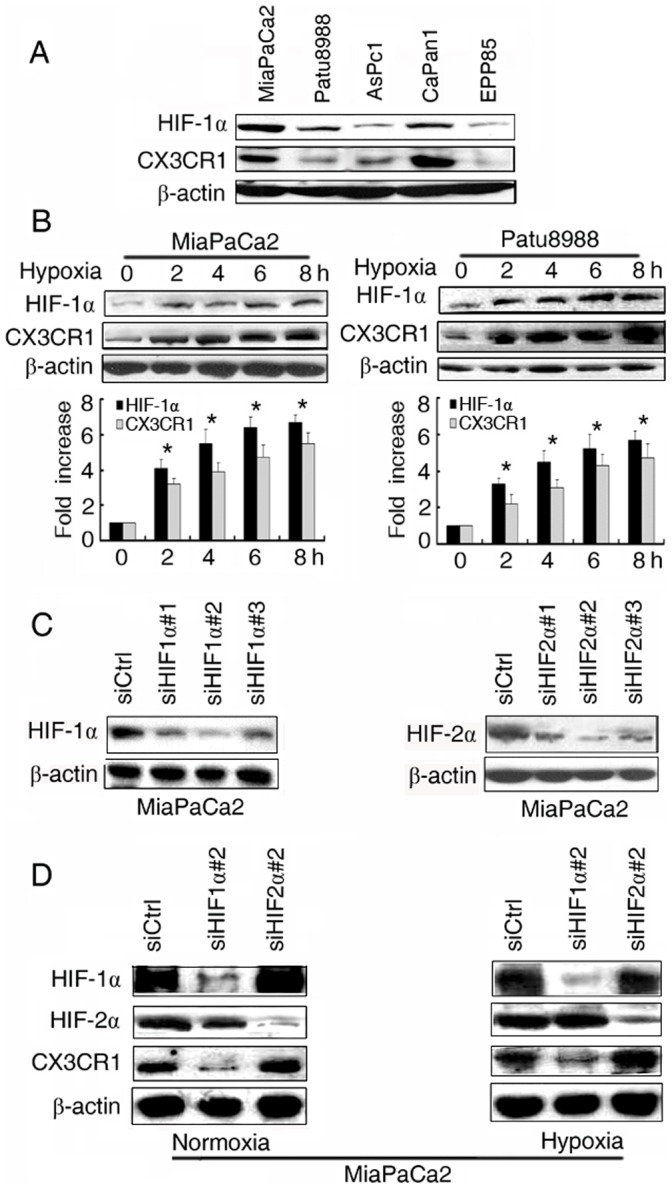
Hypoxia-induced CX3CR1 expression. (**A**) The expression of HIF-1α and CX3CR1 in five PDAC cells assessed by Western-blotting. (**B**) HIF-1α and CX3CR1 expression determined by Western-blotting (upper) and qRT-PCR (lower) on MiaPaCa2 (left) and Patu8988 (right) cells cultured under normoxia (21% O_2_) or hypoxia (1.5% O_2_) for different times. *P<0.05 versus control. (**C**) Three specific siRNAs targeting HIF-1α (left) (50 nM) and HIF-2α (right) (50 nM) on MiaPaCa2 cells. (**D**) Western-blotting for evaluating the expression of CX3CR1 after knockdown of siHIF1α #2 (50 nM) and siHIF2α #2 (50 nM) in MiaPaCa2 cells under normoxia and hypoxia conditions.

## Materials and Methods

### Cell Culture and Hypoxic Treatment

MiaPaCa2, AsPc1 and CaPan1 human PDAC cells were from the American Type Culture Collection, Patu8988 cells [14] were a gift from Prof. Shi X (Dong Nan University, Nanjing, China) and EPP85 cells [15] a gift from Prof. Zhou J (Nan Kai University, Tianjin, China). Cells were grown at 37°C in a humidified atmosphere of 95% air and 5% CO_2_, using Dulbecco’s modified Eagle media (DMEM) with 10% fetal bovine serum. For hypoxic treatment, cells were placed in a modulator incubator (Thermo Electron Co., Forma, MA) in an atmosphere consisting of 93.5% N_2_, 5.0%CO_2_ and 1.5% O_2_.

### Western Blotting Analysis

Whole-cell extracts were prepared by lysing cells with SDS lysis buffer supplemented with proteinase inhibitors cocktail (Sigma). Protein concentrations were quantified using Pierce protein assay kit. Protein lysates (20 µg) were separated by SDS-PAGE, and target proteins were detected by Western-blotting with antibodies against HIF-1α, HIF-2α, CX3CR1 and β-actin (Table S1). Specific proteins were visualized with enhanced chemiluminescence detection reagent (Pierce).

### Real-time Quantitative Reverse Transcription Polymerase Chain Reaction (qRT-PCR)

Total RNA was isolated from transfected cells by the TriPure Isolation Reagent (Roche) and used for first-strand cDNA synthesis through the First-Strand Synthesis System for reverse transcription-PCR. Then, 1 µg sample of the cDNA was quantified by real-time PCR using primer pairs with SYBR Green PCR Master mix (TaKaRa, Dalian, China). Each sample was done in triplicate. β-actin was used as loading control. PCR primers used are indicated in Table S1.

### Flow Cytometry

Cells treated with siHIF1α duplexes or pcDNA3.1-HIF1α overexpression plasmids were analyzed in an EPICS XL (Beckman Coulter) flow cytometer through FITC-labeled antibody against CX3CR1 (Biolegend).

**Figure 2:**
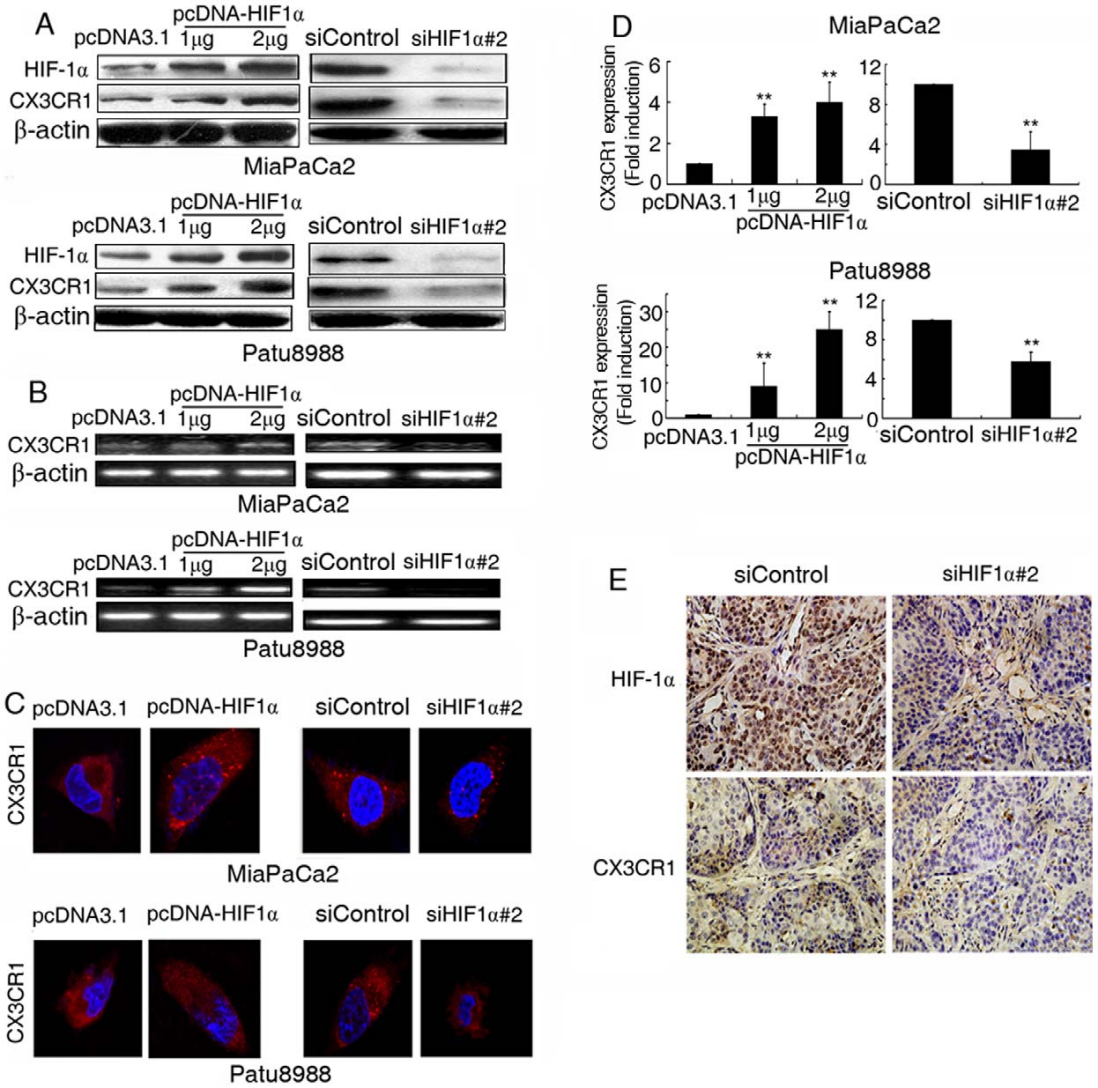
HIF-1α directly regulated the expression of CX3CR1. (**A**) MiaPaCa2 (upper) and Patu8988 (lower) cells were transfected with pcDNA3.1-HIF1α plasmids (1 µg, 2 µg) (left) or siHIF1α #2 (50 nM) (right) for 48 h and assessed by Western-blotting. (**B**) MiaPaCa2 (upper) and Patu8988 (lower) cells were transfected with pcDNA3.1-HIF1α plasmids (1 µg, 2 µg) (left) or siHIF1α #2 (50 nM) (right) for 48 h and assessed by semiquantitative PCR. (**C**) MiaPaCa2 (upper) and Patu8988 (lower) cells were transfected with pcDNA3.1-HIF1α plasmids (1 µg) (left) or siHIF1α #2 (50 nM) (right) for 48 h and assessed by confocal microscopy. Representative images for the analysis show CX3CR1 expression (in red) and nuclei (in blue) in pancreatic cancer cells. (**D**) MiaPaCa2 (upper) and Patu8988 (lower) cells were transfected with pcDNA3.1-HIF1α plasmids (1 µg, 2 µg) (left) or siHIF1α #2 (50 nM) (right) for 48 h and assessed in EPICS XL flow cytometry. Fluorescence was determined to measure the expression of CX3CR1. Value in control cells (pcDNA3.1 or siControl) were used as the baseline. Value in (pcDNA-HIF1α or siHIF1α #2) were shown as the fold induction in relation to the baseline.** P<0.01 versus control. (**E**) Immunohistochemistry of tumors originated from MiaPaCa2 cells that were transplanted in nude mice to test the effects of delivery of cholesterol-conjugated siControl (left) or siHIF1α #2 (right). (magnification: ×400).

### Confocal Microscopy

To assess HIF-1α and CX3CR1 distribution, MiaPaCa2 and Patu-8988 cells were seeded on glass slides pretreated with poly-L-lysine for different treatment. Then, cells were washed once with PBS and fixed with 4% PFA for 15 min at room temperature. After blocking with 2% bovine serum albumin and normal goat serum in PBS, cells were stained with anti-CX3CR1 (1∶350 dilution, overnight at 4°C) antibodies, followed with a DAPI staining. Coverslips were mounted with 90% glycerol, and sections examined with a confocal microscopy.

### Chromatin Immunoprecipitation Assay

A chromatin immunoprecipitation assay was performed using a commercial kit (Upstate Biotechnology) according to the manufacturer’s instruction. Primers flanking the HRE of the VEGF promoter were used as a positive control [16]. The PCR primers are indicated in Table S1 [12].

### siRNA Duplexes, Plasmid Constructs, Transient Transfection and Luciferase Assay

Small interfering RNAs (siRNAs) against HIF-1α and HIF-2α and pEGFP-C1-CX3CR1 plasmids were designed and synthesized from Ribobio (Guangzhou, China) (Table S1). pcDNA3.1-HIF-1α plasmids were prepared as previously described [16].

Genomic DNA fragments of the human CX3CR1 gene [17], spanning from +40 to −2025 relative to the transcription initiation site was generated by PCR and inserted into pGL3-Basic vectors (nominated pGL3-CX3CR1). All constructs were sequenced to confirm their identity. Luciferase activity was measured using the Dual-Luciferase Reporter Assay System (Promega) as described [16].

For transfection, cells were plated at a density of 5×10^5^ cells/well in 6-well plates containing serum-containing medium. When the cells were 80% confluent, siHIF1α duplexes or pcDNA3.1-HIF1α overexpression plasmids were transfected into cells using lipofectamine-2000 (Invitrogen) for 48 h. The cells were collected for migration analysis, Western-blotting, RT-PCR, flow cytometry and confocal microscopy.

**Figure 3:**
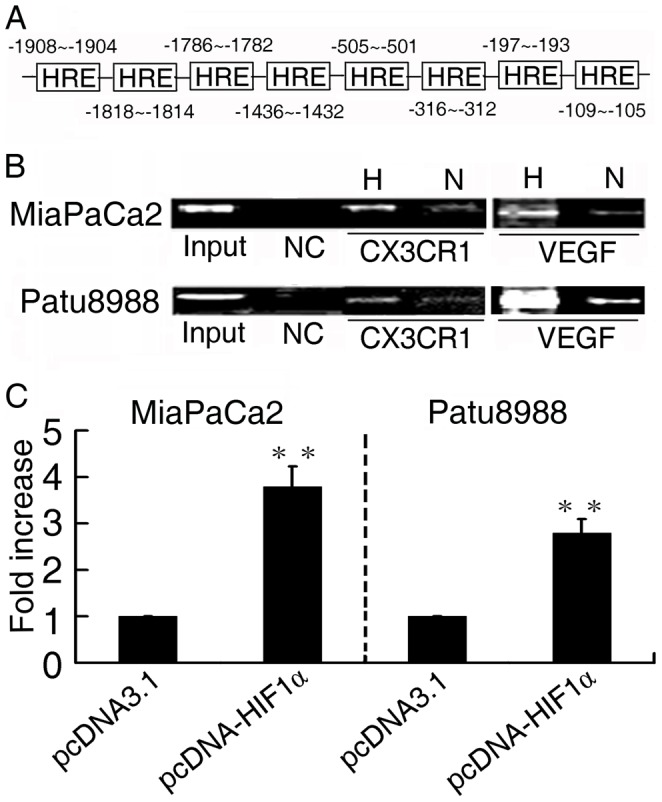
HIF-1α upregulated CX3CR1 promoter activity. (**A**) The DNA sequence of the CX3CR1 promoter. HRE sites are located at the different site. (**B**) Chromatin immunoprecipitation analysis on MiaPaCa2 (upper) and Patu8988 (lower) cells. The PCR products of VEGF promoter were used as positive control. H, N and NC indicates hypoxia, normoxia and negative control, respectively. (**C**) Luciferase analysis in MiaPaCa2 (left) and Patu8988 (right) cells. Cells were cotransfected with pGL3-CX3CR1 (1 µg) and pcDNA3.1-HIF1α plasmids (1 µg). Relative luciferase analysis was measured by Dual-Luciferase Reporter Assay System. Y axis: pGL3-CX3CR1 relative luciferase activity. ** P<0.01 versus control.

### Cell Migration Assay

SK-N-BE neuroblastoma cells were purchased from the Shanghai Cell Bank, Chinese Academy of Sciences (Shanghai, China). Cells were seeded at 10^6^ cells/mL in 6-well plates with serum-containing medium and stimulated with tumor necrosis factor α (TNFα) (20 ng/mL) and IFNγ (500 units/mL) (Peprotech). After overnight incubation, medium was replaced with serum-free medium for 24 h. The conditioned supernatants were collected [4], [18].

For transfection, cells were incubated with siHIF1α duplexes or pcDNA3.1-HIF1α overexpression plasmids for 48 h. Then, cells were rinsed with PBS and suspended in DMEM. A total of 200 µl of serum-free media containing 2.5×10^5^ cells was placed into the top of migration chambers with 8-µm filters (24-well plate format, Costar), which were standing in wells containing 700 µl of media with recombinant CX3CL1 (200 ng/ml) or containing 700 µl of SK-N-BE conditioned supernatants. The cells were incubated at 37°C for 12 h, after which the chambers were removed from the wells. Cells that had migrated to the other side of the filter bottom were fixed with 2.5% glutaraldehyde for 15 min, rinsed thoroughly with PBS, and stained with trypan blue for 5 min. Cells were counted in 10 fields (magnification, ×200). Each experiment was performed in triplicate.

**Figure 4:**
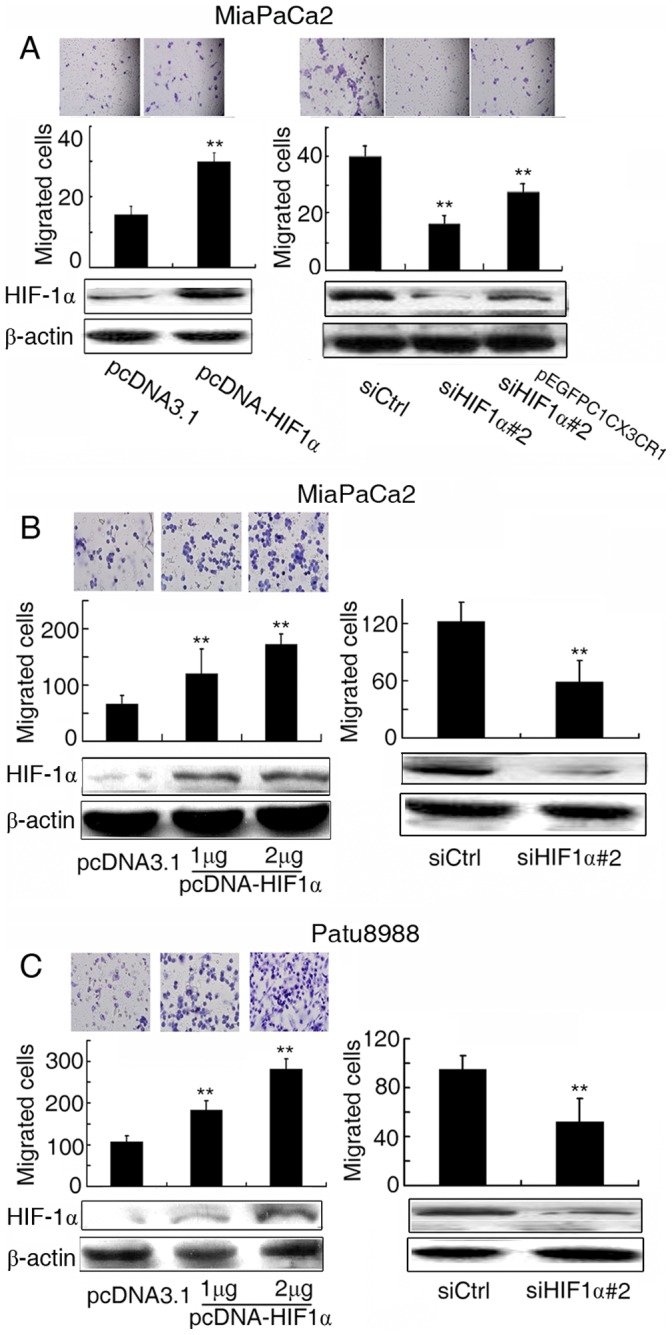
HIF-1α affected chemotactic migration of PDAC cells through CX3CR1. (**A**) MiaPaCa2 cells were cotransfected with pcDNA3.1-HIF1α plasmids (1 µg) (left) or with siHIF1α #2 (50 nM) and pEGFP-C1-CX3CR1 overexpression plasmids (1 µg) (right) for 48 h and assessed for chemotactic migration (upper) to SK-N-BE conditioned supernatants. Western-blotting (lower) was used to confirm the efficiency of siHIF1α, pcDNA3.1-HIF1α and pEGFP-C1-CX3CR1 plasmids. Migrating cells were counted under a light microscope. (magnification: ×200). ** P<0.01 versus control. (**B**) Chemotactic migration (upper) of MiaPaCa2 cells transfected with pcDNA3.1-HIF1α plasmids (1 µg, 2 µg) (left) or siHIF1α #2 (50 nM) (right) for 48 h to recombinant CX3CL1. Western-blotting (lower) was used to confirm the efficiency of siHIF1α and pcDNA3.1-HIF1α plasmids. Migrating cells were counted under a light microscope. (magnification: ×200). ** P<0.01 versus control. (**C**) Chemotactic migration (upper) of Patu8988 cells transfected with pcDNA3.1-HIF1α plasmids (1 µg, 2 µg) (left) or siHIF1α #2 (50 nM) (right) for 48 h to recombinant CX3CL1. Western-blotting (lower) was used to confirm the efficiency of siHIF1α and pcDNA3.1-HIF1α plasmids. Migrating cells were counted under a light microscope. (magnification: ×200). ** P<0.01 versus control.

### Determination of Neural Invasion in Patients and Immunohistochemistry

With an approval from local ethics committee, PDAC samples were obtained from 95 patients (ages, 36–79y) undergoing surgical resection between July 1997 and April 2010. Histologic diagnosis of PDAC was confirmed at the Tianjin Cancer Institute & Hospital. H&E slides of all the 95 cases were reviewed to evaluate the degree of neural invasion, defined as the presence of cancer cells in the perineurium of nerve fascicles as previously described [4].

**Figure 5:**
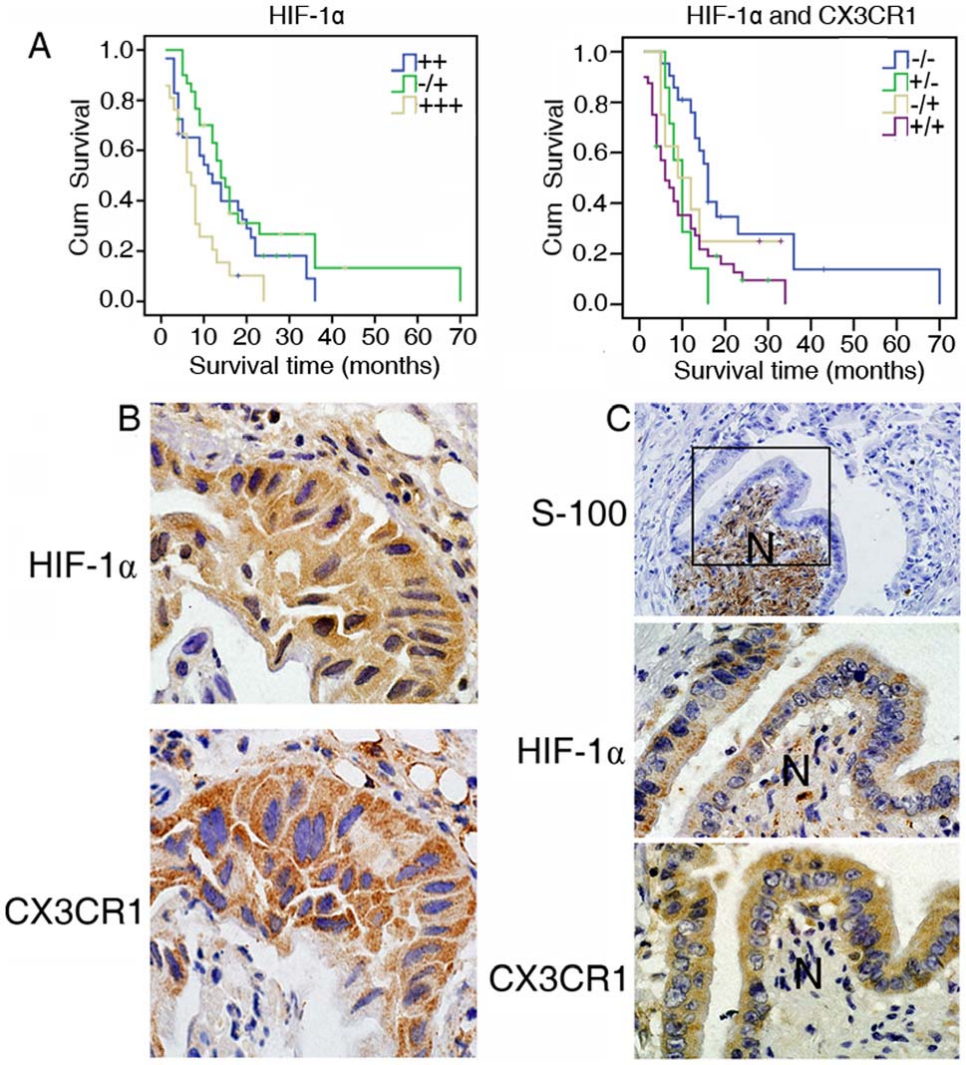
HIF-1α and CX3CR1 expression was involved in PNI of PDAC. (**A**) Overall survival by Kaplan-Meier estimates of event rates after cancer removal. PDAC patients with high positive (+++) HIF-1α protein expression had significantly worse total survival than did those who with negative or low (-or+) and medium (++) HIF-1α expressiom (left) (P<0.01). Patients with positive (+/+) HIF-1α and CX3CR1 protein expression had obviously worse survival than those with negative (HIF-1α/CX3CR1: −/−) and medium (HIF-1α/CX3CR1: −/+ or +/−) expression (right) (P<0.05). (**B**) Immunohistochemical analysis of HIF-1α (upper) and CX3CR1 (lower) correlative expression in human PDAC surgical samples. (magnification: ×1000). (**C**) High Expression of HIF-1α and CX3CR1 around nerve terminations in human PDAC surgical samples. Intratumoral nerves were stained with anti-S-100 antibody (top) (magnification: ×400). Tumor cells adjacent to intrapancreatic nerves (N) were strongly positive for HIF-1α (middle) and CX3CR1 (bottom). (magnification: ×1000).

**Table 1:** Correlation of HIF-1α expression to clinicopathological features in PDAC.

		HIF-1α				
		–	+	++	+++	*P*
Tumor size (cm)		3.81±1.95	3.50±1.53	4.49±1.88	6.45±2.86	<0.01
Histological grade						>0.05
G1		8	9	13	9	
G2		5	4	8	4	
G3		3	6	16	10	
LN metastasis						<0.05
N0		10	9	13	6	
N1		5	10	23	15	
pTNM stage						<0.01
I		2	2	1	0	
II		11	11	18	6	
III		0	5	9	8	
IV		2	1	8	7	
PNI						<0.01
–		13	5	7	3	
+		3	14	30	20	
CX3CR1						<0.01
–		11	5	1	2	
+		3	0	3	1	
++		1	10	17	3	
+++		1	3	14	17	

The statistical data of HIF-1 and CX3CR1 expression on clinicopathological features in PDAC surgical samples. *P* values were calculated by Spearman’s rank-correlation test;tumor size (cm): Expressed as mean, *F* = 7.81, *P*<0.01 (anova test). N0 and N1 refer to the absence and presence of regional lymph node (LN) metastasis, respectively. pTNM stage refers to the pathological tumor node metastasis (pTNM) stage.The numbers of samples vary because clinical data were incomplete in some cases (i: For the cases that lymph node metastasis was known, ii: For the cases that pathological tumor node metastasis stage was known, iii: For the cases that CX3CR1 was known).

Paraffin-embedded pancreatic tissue sections were deparaffinized and rehydrated with xylene and graded alcohols. Antigen retrieval was carried out in 5 mM citrate buffer. After inactivation of endogenous peroxidase with 3% H_2_O_2_, sections were blocked with goat serum and incubated with anti-HIF-1α (1∶100 dilution), anti-CX3CR1 (1∶350 dilution) or anti-S-100 antibodies (overnight at 4°C) (Table S1). The sections were then incubated with biotinylated secondary antibody and streptavidin biotin-peroxidase. Diaminobenzidine was used as a chromogen substrate. Finally, the sections were counterstained with haematoxylin. Both examiners were blinded to clinicopathologic data. Immunoreactivity was scored semiquantitatively according to the percentage of positive tumor cells as described previously [4]. Intensity of staining was scored as (0 = negative; 1 = low; 2 = medium; 3 = high). Eight random highpower fields (magnification: ×400) were observed under a light microscope and the percentage of positive cells in four high-power fields were recorded. Extent of staining was scored as 0 = 0% stained; 1 = 1–25% stained; 2 = 26–50% stained; 3 = 51–100% stained. The final score was determined by multiplying the scores of intensity with the extent of staining, ranging 0–9. Final scores (intensity score × percentage score) of less than 2 were considered as negative staining (−), 2–4 as low staining (+), 4–6 as medium staining (++) and >6 as high staining (+++). HIF-1α and CX3CR1 protein expression was defined as negative (–) when the scores were between (0–4) and positive (+) when the scores were above 4.

### In vivo Assay

All animal experiments were undertaken in accordance with the National Institute of Health Guide for the Care and Use of Laboratory Animals, with the approval of Tianjin Cancer Institute & Hospital. For preparation of subcutaneous model, MiaPaCa2 cells (5×10^5^) were injected subcutaneously into the right flanks of female nude mice (eight mice per group). For delivery of cholesterol-conjugated siHIF-1α, 10 nmol RNA in 0.1 ml saline buffer was injected into tumor mass once every 3 days for 3 weeks [19]. Then the mice were sacrificed and the tumor was removed, fixed in 10% formalin, and embedded in paraffin. The samples were processed for histologic examination and stained with anti-HIF-1α (1∶100 dilution), anti-CX3CR1 (1∶350 dilution) antibodies overnight at 4°C [4].

### Statistical Analysis

All clinical characteristics were compared by the χ^2^ test for categorical variables. Student’s t test or ANOVA for unpaired data was used to compare mean values. Spearman’s rank correlation coefficient test was carried out for testing the association between ordinal variables. All probability values were two sided. Analyses were performed using the SPSS13.0 statistical analysis software. Each experiment was done in triplicate and values are presented as mean±SD, unless otherwise stated.

## Results

### Hypoxia-induced CX3CR1 Expression

HIF-1α is constitutively expressed in PDAC. Human PDAC cell lines were tested for the expression of HIF-1α and CX3CR1 proteins. Western-blotting analysis showed that the expression of HIF-1α protein was in parallel with that of CX3CR1 protein in PDAC cell lines (Figure 1A).

Since hypoxia is a common feature of the microenvironment of solid tumors, it is important to detect whether hypoxia promotes the expression of CX3CR1 in pancreatic cancer cells. CX3CR1 expression was determined by Western-blotting on MiaPaCa2 and Patu8988 cells cultured under normoxia (21% O_2_) or hypoxia (1.5% O_2_) for different times. The hypoxic condition was defined to mimic the average oxygen tension in tumors reported previously [20]. As shown in Figure 1B, compared with normoxia, CX3CR1 expression (protein and mRNA) was increased after hypoxic treatment.

To identify the function of HIF-1α and HIF-2α in hypoxia-induced expression of CX3CR1 in pancreatic cancer, we used three specific siRNAs targeting HIF-1α or HIF-2α and all of them effectively reduced HIF-1α or HIF-2α expression (Figure 1C). We found that knockdown of HIF-1α expression dramatically decreased the expression of CX3CR1 but did not find the decrease after silence of HIF-2α (Figure 1D), suggesting that HIF-1α plays a critical role for the expression of CX3CR1.

### HIF-1α Regulated the Expression of CX3CR1 in vitro and in vivo

To determine the roles of HIF-1α in the expression of CX3CR1, we up-regulated the expression of HIF-1α by tansfecting pcDNA3.1-HIF1α plasmids. Following the overexpression of HIF-1α, CX3CR1 protein and mRNA expression and CX3CR1 distribution were markedly increased (Figures 2A, B, C, D,left, *P*<0.01). In contrast, repression of HIF-1α notably decreased the expression of CX3CR1 (Figures 2A, B, C, D,right, *P*<0.01).

To investigate in vivo the correlation of HIF-1α and CX3CR1 in pancreatic cancer, MiaPaCa2 cells were injected subcutaneously into nude mice. When tumors were treated with cholesterol-conjugated siHIF-1α, HIF-1α and CX3CR1 expressions were decreased (Figure 2E,*P*<0.01). The results suggest that HIF-1α and CX3CR1 are correlated in vivo.

### HIF-1α Upregulated CX3CR1 Promoter Activity

Having shown that HIF-1α plays a critical role in CX3CR1 expression, we further investigated whether HIF-1α directly regulated CX3CR1 gene. Screening of the 5′-flanking region of the CX3CR1 gene revealed eight potential HIF-1-binding sites (Figure 3A). To demonstrate the binding of HIF-1α to the CX3CR1 promoter, chromatin immunoprecipitation assay was performed in MiaPaCa2 (Figure 3B, upper) and Patu8988 (Figure 3B, lower) cells at 1.5% O_2_ or 21% O_2_. In chromatin fractions pulled down by an anti-HIF-1α antibody, CX3CR1 promoter was detected. These fragments were significantly increased (*P*<0.01) under hypoxia. However, CX3CR1 promoter was not found in samples pulled down by a control IgG antibody.

In order to determine whether the binding of HIF-1α to the CX3CR1 promoter could activate the promoter, we constructed the full-length CX3CR1 luciferase promoter vector and transfected it with HIF-1α cDNA fragment into MiaPaCa2 and Patu8988 cells. Luciferase analysis showed that overexpression of HIF-1α (pcDNA-HIF1α) markedly increased the CX3CR1 promoter activity in MiaPaCa2 (∼3.8-fold, *P*<0.01) and Patu8988 cells (∼2.8-fold, *P*<0.01) compared with control vectors (pcDNA3.1) (Figure 3C).

### HIF-1α Affected the Chemotactic Migration of PDAC Cells through CX3CR1

To explore the roles of HIF-1α in migration of PDAC cells in vitro, we performed chemotactic migration assays as described previously [4]. Overexpression of HIF-1α increased the chemotactic migration of MiaPaCa2 cells toward SK-N-BE conditioned supernatants (Figure 4A, left, *P*<0.01). Repression of HIF-1α decreased the chemotactic migration of MiaPaCa2 cells (Figure 4A, right, *P*<0.01).

It has been reported that CX3CR1 mediated chemotactic migration toward CX3CL1 gradients. We further detected that overexpression of HIF-1α increased the chemotactic migration of MiaPaCa2 (Figure 4B, left) and Patu8988 (Figure 4C, left) cells toward recombinant CX3CL1 (*P*<0.01). Repression of HIF-1α decreased the chemotactic migration of MiaPaCa2 (Figure 4B, right) and Patu8988 (Figure 4C, right) cells toward recombinant CX3CL1 (*P*<0.01). Taken together, both loss-of-function and gain-of-function studies demonstrated that HIF-1α is involved in the chemotactic migration of PDAC cells in vitro.

To verify the roles of CX3CR1 in HIF-1α mediated chemotactic migration, we co-transfected siHIF1α duplexes and pEGFP-C1-CX3CR1 plasmids into MiaPaCa2 cells. As shown in Figure 4A (right), overexpression of CX3CR1 reversed the inhibitory effect of HIF-1α knockdown on chemotactic migration of PDAC cells to the SK-N-BE conditioned supernatants (*P*<0.01), suggesting that CX3CR1 is involved in the chemotactic migration mediated by HIF-1α.

### HIF-1α and CX3CR1 Expression was Involved in Perineural Invasion (PNI) of PDAC

We assessed the expression of HIF-1α in PDAC samples by immunohistochemistry and found that HIF-1α was correlated with tumor diameter (*F* = 7.81, *P*<0.01), lymph node metastasis (*r* = 0.24, *P*<0.05), the pathological tumor node metastasis stage (*r* = 0.39, *P*<0.01) and PNI (*r* = 0.43, *P*<0.01) of PDAC samples (Table 1). PDAC patients with high positive (+++) HIF-1α protein expression had significantly worse total survival than those with negative or low (-or+) and medium (++) HIF-1α expression (*P*<0.01) (Figure 5A, left). Furthermore, patients with positive (+/+) HIF-1α and CX3CR1 protein expression had obviously worse survival than those with negative (HIF-1α/CX3CR1: −/−) and medium (HIF-1α/CX3CR1: −/+ or +/−) expression (*P*<0.05) (Figure 5A, right).

CX3CR1 has been identified as an important factor for PNI [4]. Immunohistochemistry that evaluated the relationship between HIF-1α and CX3CR1 showed that CX3CR1 expression was correlated with HIF-1α in PDAC surgical samples (Figure 5B and Table 1, *r* = 0.58, *P*<0.01).

Nerve terminations were surrounded by tumor cells with high expression of HIF-1α and CX3CR1 (Figure 5C). Hence, HIF-1α and CX3CR1 expression is strongly associated with more PNI in pancreatic cancer patients.

## Discussion

In this study, we have confirmed that CX3CR1 was highly expressed in both clinical samples and cell lines of pancreatic cancer and that hypoxia induced the expression of CX3CR1. Furthermore, small interfering RNAs (siRNAs) analysis revealed that HIF-1α, but not HIF-2α, influenced the hypoxia-induced expression of CX3CR1 in vitro and in vivo. Meanwhile, overexpression of HIF-1α protein upregulated the expression of CX3CR1 in pancreatic cancer cells.

As a transcription factor, HIF-1 exerts its biological function through activating target genes. By chromatin immunoprecipitation and luciferase analysis, we showed evidence that HIF-1α bound to the HREs region of CX3CR1 promoter and upregulated the transcription. It has been demonstrated that CX3CR1 could regulate migration and PNI of pancreatic cancer cells [4]. Both gain-of-function and loss-of-function studies showed that HIF-1α affected the chemotactic migration of PDAC cells toward supernatants of activated neuroblastoma (conditioned supernatants) and CX3CL1. Importantly, overexpression of CX3CR1 reversed the inhibitory effect of siHIF-1α on PDAC cell migration to the conditioned supernatants, suggesting that HIF-1α influences the migration of PDAC cells by CX3CR1.

The CX3CR1 receptor has been shown to influence the prognosis of PDAC patients. In our study, clinicopathological and experimental data uncovered the co-expression of HIF and CX3CR1 around nerve tissues in PDAC. Although PNI is a key pathological feature of PDAC relating to recurrence, little is known about its mechanism. Previous investigations were limited to the cytokines and their receptors for the interaction between cancer cells and nerve cells. In our opinion, it is more significant to uncover the mechanism of chemotactic migration at transcriptional levels because the transcription factor represents the mutual pathways for multiple effectors. HIF-1 might be a key transcription factor to influence chemotactic migration and could be an effective target to prevent the recurrence of PDAC.

Previous studies showed that HIF-1 was constitutively expressed in pancreatic cancer. In deed, intratumoral hypoxia and oncogene mutation (such as Akt or MAPK pathways) induce the expression of HIF-1α. HIF-1α expression in PDAC cells is heterogeneous. So, different PDAC cells have various extents of chemotactic migration. Recent evidence has verified that CX3CL1 upregulated the expression of HIF-1α in human aortic endothelial cells [21]. We also found that recombinant CX3CL1 enhanced the expression of HIF-1α in PDAC cell lines (Figure S1). It is reasonable that CX3CL1/CX3CR1 might contribute to the constitutive expression of HIF-1α in PDAC. Thus, The crosstalk between HIF-1 and CX3CR1 may form a positive feed-back loop for maintaining the capability of chemotactic migration in PDAC.

CX3CR1 was expressed by dendritic cells, natural killer cells, macrophages and monocytes and was associated with immune-related diseases. According to our findings, HIF-1 may also mediate the expression of CX3CR1 in immune cells and be involved in hypoxia-related inflamatory response. Nowadays, many chemical compounds are available as inhibitors of HIF-1 [22]. We suggest that inhibiting HIF-1 expression may be more effective for the treatment of invasive PDAC compared with targeting a single chemokine.

## Supporting Information

Figure S1 **MiaPaCa2 (left) and Patu-8988 (right) cells were treated with CX3CL1 (200** **ng/ml) for different times and then were lysed for western-blotting.**
(TIF)

Table S1 **The antibodies, siRNAs and primer sequences in the experiment.**
(DOC)

## References

[1] Imai T, Hieshima K, Haskell C, Baba M, Nagira M, et al (1997) Identification and molecular characterization of fractalkine receptor CX3CR1, which mediates both leukocyte migration and adhesion. Cell 91: 521–530. 9390561 10.1016/s0092-8674(00)80438-9

[2] Shulby SA, Dolloff NG, Stearns ME, Meucci O, Fatatis A (2004) CX3CR1-fractalkine expression regulates cellular mechanisms involved in adhesion, migration, and survival of human prostate cancer cells. Cancer Res 64: 4693–4698. 15256432 10.1158/0008-5472.CAN-03-3437

[3] Andre F, Cabioglu N, Assi H, Sabourin JC, Delaloge S, et al (2006) Expression of chemokine receptors predicts the site of metastatic relapse in patients with axillary node positive primary breast cancer. Ann Oncol 17: 945–951. 16627550 10.1093/annonc/mdl053

[4] Marchesi F, Piemonti L, Fedele G, Destro A, Roncalli M, et al (2008) The chemokine receptor CX3CR1 is involved in the neural tropism and malignant behavior of pancreatic ductal adenocarcinoma. Cancer Res 68: 9060–9069. 18974152 10.1158/0008-5472.CAN-08-1810

[5] Bazan JF, Bacon KB, Hardiman G, Wang W, Soo K, et al (1997) A new class of membrane-bound chemokine with a CX3C motif. Nature 385: 640–644. 9024663 10.1038/385640a0

[6] Pan Y, Lloyd C, Zhou H, Dolich S, Deeds J, et al (1997) Neurotactin, a membrane-anchored chemokine upregulated in brain inflammation. Nature 387: 611–617. 9177350 10.1038/42491

[7] Haskell CA, Cleary MD, Charo IF (2000) Unique role of the chemokine domain of fractalkine in cell capture. Kinetics of receptor dissociation correlate with cell adhesion. J Biol Chem 275: 34183–34189. 10940307 10.1074/jbc.M005731200

[8] Verge GM, Milligan ED, Maier SF, Watkins LR, Naeve GS, et al (2004) Fractalkine (CX3CL1) and fractalkine receptor (CX3CR1) distribution in spinal cord and dorsal root ganglia under basal and neuropathic pain conditions. Eur J Neurosci 20: 1150–1160. 15341587 10.1111/j.1460-9568.2004.03593.x

[9] Rebecca S, Deepa N, Ahmedin J (2012) Cancer statistics, 2012. CA Cancer J Clin 62: 10–29.22237781

[10] Gao YJ, Ji RR (2010) Chemokines, neuronal-glial interactions, and central processing of neuropathic pain. Pharmacol Ther 126: 56–68. 20117131 10.1016/j.pharmthera.2010.01.002 PMC2839017

[11] Pour PM, Bell RH, Batra SK (2003) Neural invasion in the staging of pancreatic cancer. Pancreas 26: 322–325. 12717262 10.1097/00006676-200305000-00002

[12] Hung SC, Pochampally RR, Hsu SC, Sanchez C, Chen SC, et al (2007) Short-term exposure of multipotent stromal cells to low oxygen increases their expression of CX3CR1 and CXCR4 and their engraftment in vivo. PLoS One 2: e416. 17476338 10.1371/journal.pone.0000416 PMC1855077

[13] Mimura I, Tanaka T, Wada Y, Kodama T, Nangaku M (2011) Pathophysiological response to hypoxia - from the molecular mechanisms of malady to drug discovery: epigenetic regulation of the hypoxic response via hypoxia-inducible factor and histone modifying enzymes. J Pharmacol Sci 115: 453–458. 21422728 10.1254/jphs.10r19fm

[14] Elsässer HP, Lehr U, Agricola B, Kern HF (1992) Establishment and characterisation of two cell lines with different grade of differentiation one primary human pancreatic adenocarcinoma. Virchows Arch B Cell Pathol Incl Mol Pathol 61: 295–306. 1348891 10.1007/BF02890431

[15] Liu M, Wang X, Yang Y, Li D, Ren H, et al (2010) Ectopic expression of the microtubule-dependent motor protein Eg5 promotes pancreatictumourigenesis. J Pathol. 221: 221–228. 10.1002/path.2706 20455257

[16] Han ZB, Ren H, Zhao H, Chi Y, Chen K, et al (2008) Hypoxia-inducible factor (HIF)-1 alpha directly enhances the transcriptional activity of stem cell factor (SCF) in response to hypoxia and epidermal growth factor (EGF). Carcinogenesis 29: 1853–1861. 18339685 10.1093/carcin/bgn066

[17] Garin A, Pellet P, Deterre P, Debré P, Combadière C (2002) Cloning and functional characterization of the human fractalkine receptor promoter regions. Biochem J 368: 753–760. 12234253 10.1042/BJ20020951 PMC1223041

[18] Fraticelli P, Sironi M, Bianchi G, D’Ambrosio D, Albanesi C, et al (2001) Fractalkine (CX3CL1) as an amplification circuit of polarized Th1 responses. J Clin Invest 107: 1173–1181. 11342581 10.1172/JCI11517 PMC209276

[19] Hou J, Lin L, Zhou W, Wang Z, Ding G, et al (2011) Identification of miRNomes in human liver and hepatocellular carcinoma reveals miR-199a/b-3p as therapeutic target for hepatocellular carcinoma. Cancer Cell 19: 232–243. 21316602 10.1016/j.ccr.2011.01.001

[20] Vaupel P, Kelleher DK, Höckel M (2001) Oxygen status of malignant tumors: pathogenesis of hypoxia and significance for tumor therapy. Semin. Oncol 28: 29–35. 10.1016/s0093-7754(01)90210-6 11395850

[21] Ryu J, Lee CW, Hong KH, Shin JA, Lim SH, et al (2008) Activation of fractalkine/CX3CR1 by vascular endothelial cells induces angiogenesis through VEGF-A/KDR and reverses hindlimb ischaemia. Cardiovasc Res 78: 333–340. 18006432 10.1093/cvr/cvm067

[22] Semenza GL (2010) Defining the role of hypoxia-inducible factor 1 in cancer biology and therapeutics. Oncogene 29: 625–634. 19946328 10.1038/onc.2009.441 PMC2969168

