# Supplementary figures and images for: Hypoxia-Inducible Factor-1α Regulates Chemotactic Migration of Pancreatic Ductal Adenocarcinoma Cells through Directly Transactivating the CX3CR1 Gene
Source: PLoS One. 2012 Aug 27;7(8):e43399. doi: 10.1371/journal.pone.0043399 (PMC3428361; doi:10.1371/journal.pone.0043399)

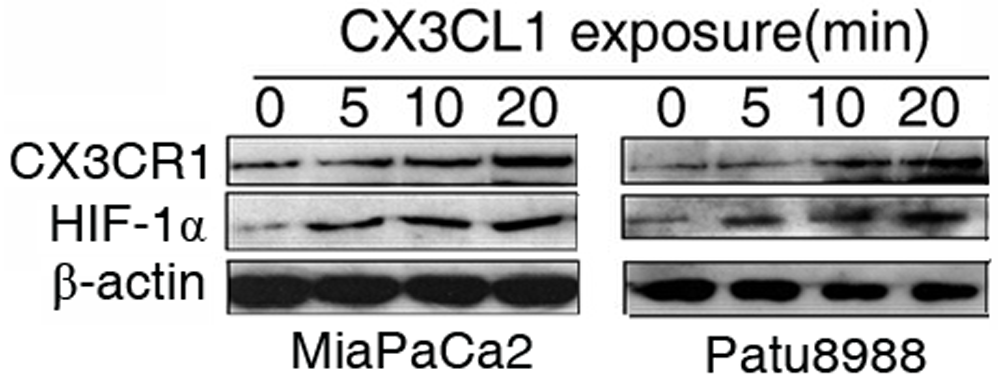

Supplement: Figure S1 — MiaPaCa2 (left) and Patu-8988 (right) cells were treated with CX3CL1 (200 ng/ml) for different times and then were lysed for western-blotting. (TIF) [file pone.0043399.s001.tif]
