# Supplementary material for: Hypoxia-Inducible Factor-1α Regulates Chemotactic Migration of Pancreatic Ductal Adenocarcinoma Cells through Directly Transactivating the CX3CR1 Gene
Source: PLoS One. 2012 Aug 27;7(8):e43399. doi: 10.1371/journal.pone.0043399 (PMC3428361; doi:10.1371/journal.pone.0043399)
Supplement: Table S1 — The antibodies, siRNAs and primer sequences in the experiment. (DOC) [file pone.0043399.s002.doc]

**Supporting Information**

**Table S1. The antibodies, siRNAs and primer sequences in the experiment**

| *Antibodies* | | | | | | |
| --- | --- | --- | --- | --- | --- | --- |
| Name | | Manufacturer | Number | Type | Usage | |
| HIF-1α | | Santa Cruz | sc-13515 | Monoclonal | WB | |
|  | |  | sc-10790 | Polyclonal | IHC | |
| HIF-2α | | Santa Cruz | sc-46691 | Monoclonal | WB | |
| CX3CR1 | | Abcam | ab8021 | Polyclonal | WB,IHC,IF | |
| S-100 | | Maixin | MAB-0585 | Monoclonal | IHC | |
| CX3CR1-FITC | | Biolegend | 341606 |  | FC | |
| *siRNAs* | | *Target sequences* | | | | |
| HIF1α #1 | | CTGATGACCAGCAACTTGA | | | | |
| HIF1α #2 | | CAATCAAGAAGTTGCATTA | | | | |
| HIF1α #3 | | GCACAGTTACAGTATTCCA | | | | |
| HIF2α #1 | | GCCCGGATAGACTTATTGC | | | | |
| HIF2α #2 | | CAGCATCTTTGATAGCAGT | | | | |
| HIF2α #3 | | CAGCAUCUUUGAUAGCAGU | | | | |
| *Primer sequences* | | | | | | |
| CX3CR1-P* | ATTCAGCAGATATAGGGCAG/ ACAGTCAGCTCTCATTAATG | | | | | ChIP |
| VEGF-P* | GCCTCTGTCTGCCCAGCTGC/ GTGGAGCTGAGAACGGGAAGC | | | | | ChIP |
| HIF1α | GCAAGCCCTGAAAGCG/ GGCTGTCCGACTTTGA | | | | | PCR |
| CX3CR1 | AGAGTGTCACCGACATTTACCTC/ GAAGAAGAAGGCGGTAGTGAA | | | | | PCR |
| β-actin | CAGAGCAAGAGAGGCATCC / CTGGGGTGTTGAAGGTCTC | | | | | PCR |

WB: Western blotting; IHC: Immunohistochemistry; IF: Immunofluorescence; FC: Flow cytometry; siRNA: Small interfering RNA; P*: Promoter
